# Notes on a Case of Fracture of the Superior Maxillæ

**Published:** 1892-04

**Authors:** 


ARTICLE VII.

### NOTES ON A CASE OF FRACTURE OF THE SUPERIOR MAXILLÆ.

Mr. F. Page said the patient was admitted into Leith Hospital on the 13th September, 1891, with a history of having been kicked on the face by a horse.

On admission, patient was suffering from the effects of alcohol, and on examination the soft parts over the face were found bruised and swollen, and the upper lip torn through in the middle line. The cartilaginous septum of the nose was torn and displaced, the wounded organ being somewhat carried to one side, and tilted up. Corresponding to the split in the lip was a fracture almost in the middle line of the jaws, from which branched off, half an inch up from the alveolar margin, another fracture through the left maxilla, which ran obliquely towards the outer angle of the eye. Passing off at the same place, but on the right side, a fracture could be traced running upwards towards the inner angle of the eye, and another at right angles to this, running outwards a quarter of an inch below the orbital plate, and extending backwards. The fractured parts dropped, and were pushed backwards, so much so, that on the right side the posterior cusps of the wisdom teeth alone impinged—the patient being unable to close his mouth, there being an interval of about half an inch between the upper and lower teeth in front. The whole body of the superior maxillæ seemed detached from the bones of the skull, and could be easily pulled forward.

About two hours after admission, the patient complained of something choking him, and on looking into the mouth, the right tonsil was seen to be much enlarged, and quickly increasing in size, threatening suffocation. An incision was made into the tonsil, and blood ran freely from it, the patient being turned on his face to prevent choking. This was followed by immediate relief, the swelling proving to be effusion into the loose tissue of the soft palate. Hæmorrhage was for a time free, but by means of cold and pressure, was ultimately controlled.

In two days, the patient now being comparatively comfortable, it was thought necessary that something should be done to correct the deformity, but on examination the fractured portions were found so intensely painful to the touch, that it was decided to wait some little time before attempting any correction.

Impressions were taken five days after the accident, great difficulty being experienced—though everything was done to gain the confidence of the patient—the whole body of the superior maxillæ having to be raised to gain insertion of tray and modelling composition. Fortunately the first attempt produced impressions deemed sufficient upon which to build an inter-dental splint.

The apparatus shown was designed, and on September the 20th it was applied. The patient was given chloroform, but this had to be abandoned, as symptoms of asphyxia were threatening, and the operation had to be completed with the patient in a semi-conscious condition. The splint was jumped into its place, and was found all that could be desired, the struggles of the patient alone tending to any difficulty in correcting the deformity. Following this, Petit's tourniquet was used, and applied round the vertex of the chin, in order that pressure might be gradually exerted on the lower jaw. As shown, by means of this tourniquet gradual pressure could be made by tightening the screw of the instrument, the band, however, by compressing the superficial veins, interfering somewhat with the venous return from the face, giving the patient a cyanosed appearance whenever the band was tightened. This was obviated to a considerable degree by pads of corrosive wool applied underneath the band.

Patient was fed through the opening in the apparatus with fluid foods, and in a few days was able, occasionally, to smoke his pipe comfortably, the only discomfort being caused by the continual tightening of the screw.

The lower jaw was kept continually pressing against the splint until October the 22nd, something like five weeks after the insertion. The apparatus, which had not been touched since its insertion, was then removed, and the result gave the utmost satisfaction, one or two cusps of teeth alone preventing perfect articulation.

In a few days the patient had ground these down himself, and in a short time left Leith Hospital with little or no visible signs of the accident.—*Dental Record.*

